# The Surgical Management of Holospinal Epidural Abscess: A Case Report and Review on Catheter-Based Irrigation Techniques

**DOI:** 10.7759/cureus.30437

**Published:** 2022-10-18

**Authors:** Dia R Halalmeh, Jeni Page, Christopher Childers, Marc D Moisi

**Affiliations:** 1 Neurosurgery, Hurley Medical Center, Flint, USA; 2 Neurosurgery, Texas Tech University Health Sciences Center, Lubbock, USA

**Keywords:** irrigation, holospinal, epidural, decompression, catheter, abscess

## Abstract

Holospinal epidural abscess (HEA) is an extremely rare spinal infection involving the entire spine and is infrequently reported in the literature. Cases with evidence of spinal cord compression and consequent neurological deficit are typically managed with prompt surgical drainage and broad-spectrum antibiotics. Surgical intervention is often challenging because this condition is inherently associated with poor prognosis and serious complications, including death. During the surgical evacuation of the abscess, catheter-based irrigation must be adequately performed. In the majority of reported cases, the extent of the advancement of the epidural catheter is blindly assessed by the operating surgeon, increasing the risk of residual collections and subsequent persistent infection. Herein, we report a rare case of HEA that was successfully treated with surgical evacuation and skip laminectomies. We also describe a catheter-based technique that facilitates adequate irrigation, thereby ensuring the complete drainage of HEA in obscured perispinal areas, as well as the decompression of the spinal cord. Postoperative neurological examination exhibited marked improvement in motor function (compared with a baseline of complete quadriparesis), indicating the successful decompression of the spinal cord and neurological improvement.

## Introduction

Holospinal epidural abscess (HEA) is an extremely rare infection of the spine. It is characterized by extensive involvement of the spinal cord from C1 to the sacrum. Generally, the involvement of the four main regions of the spine (cervical, thoracic, lumbar, and sacral) is considered holospinal. Given the scarcity of literature on this condition, the incidence of HEA cannot be accurately determined. However, in a meta-analysis of 915 patients with spinal epidural abscesses, only 1% developed HEA [1]. An estimated mortality rate of more than 15% has been reported in the literature [2]. Surgical decompression with intravenous broad-spectrum antibiotics remains the preferred management by most authors [1,3-7], especially in patients who develop neurological deficits. Accordingly, multiple and spaced decompression sites, in a procedure called “skip laminectomies,” are usually performed and appear to be a safe and effective approach in such cases.

Importantly, catheter-based drainage with the irrigation and aspiration of purulence is necessary for the resolution of the abscess. The terminal point of catheter advancement in the epidural space is usually difficult to assess, and the process of surgical drainage is therefore a blind one, especially with extensive abscesses such as HEA. This may increase the risk of inadequate irrigation and incomplete evacuation of the abscess in perispinal distant spaces where surgical access can be difficult or overlooked. Consequently, the recurrence of the infection from these sites may occur. In this report, we present a rare case of HEA and describe a catheter-based technique that allows for the identification of the catheter tip after full advancement while maintaining sufficient evacuation and decompression of the spinal cord.

## Case presentation

A 48-year-old male patient with a history of poorly controlled diabetes was brought to the emergency department due to inability to move his extremities. His symptoms progressively worsened four days prior to presentation. The patient was recently diagnosed with left-sided osteomyelitis and discitis at L3-L4 levels with extensive amounts of purulence involving the iliopsoas and paraspinal muscles. His temperature was 38.6°C (101.5°F), blood pressure was 117/86 mm Hg, pulse was 68/minute, and respirations were 14/minute. Complete blood count revealed high white blood cell count, high platelet count, and low-normal hemoglobin. A review on the laboratory results is summarized in Table 1.

**Table 1:** Summary of laboratory results at presentation CBC: complete blood count; WBC: white blood cell; Hb: hemoglobin; PLT: platelet; K/UL: thousands per cubic milliliter

Laboratory test	Value	Reference range and units
CBC		
WBC count	35.2 K/UL	4.0-10.8 K/UL
Neutrophils	93%	36%-75%
Bands	11%	0%-3%
Lymphocytes	3%	20%-50%
Hb	13.0 g/dL	13.5-17.5 g/dL
PLT count	555.0 K/UL	130-430 K/UL
Glucose	167 mg/dL	64-90 mg/dL

**Figure 1:**
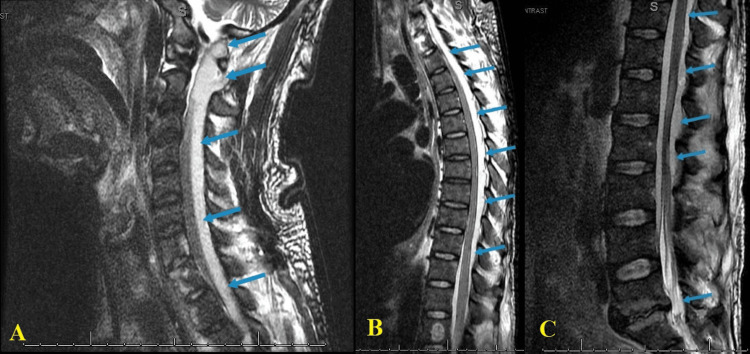
Holospinal epidural abscess T2-weighted sagittal MRI of the (a) cervical, (b) thoracic, and (c) lumbosacral spine demonstrating posterior, peripherally enhancing intraspinal epidural abscess (blue arrows) extending from the base of the skull to the sacrum (past the inferior margin of the scan: S1) MRI: magnetic resonance imaging

Motor strength was 0/5 in the upper and lower extremities, and deep tendon reflexes were absent throughout. The patient also reported urinary incontinence. Emergent magnetic resonance imaging (MRI) with and without contrast of the spine revealed a diffuse, posterior, peripherally enhancing epidural abscess extending from the base of the skull to the sacrum with evidence of compression and mass effect (Figure 1).

Given the risk of lethal septic shock, empiric broad-spectrum antibiotics were started immediately. Urine and blood cultures were taken before the initiation of empiric antibiotics and later showed evidence of *Staphylococcus aureus* growth. Based on his neurological deficit and radiological findings, he was taken to the operating room for evacuation and spinal cord decompression. Informed consent was obtained from the patient himself.

Operative technique

The patient was positioned prone on a radiolucent table after intubation. Target levels for laminectomies were identified using intraoperative fluoroscopic (C-arm) guidance. The skin was marked for four separate incisions: cervical (C4-C5), midthoracic (T5-T6), lower thoracic (T11-T12), and lumbar (L2-L4) incisions. The mass effect caused by the HEA was most notable at these levels. Junctional areas/vertebrae were avoided to prevent the destabilization of the spine and later potential deformities. A single laminectomy was performed through each incision except for the lumbar region where two contiguous laminectomies were carried out (L2-L4) due to the presence of phlegmon near these levels, as well as the deterioration of L3-L4 facet joints (see below). Upon the removal of the ligamentum flavum and laminae, a copious amount of purulence was encountered under high pressure, and irrigation with normal saline using an external ventricular drain (EVD) catheter (inner diameter: 1.5 mm; outer diameter: 2.8 mm) was performed. To ensure adequate irrigation after performing laminectomies, the EVD catheter was tunneled epidurally through the incisions in the following manner.

Cervical Incision

Initially, the catheter was advanced through the cervical incision cranially (toward the base of the skull) and caudally (toward the midthoracic incision) with concurrent irrigation with normal saline. Once it was fully advanced, the catheter was pulled out slowly while continuing irrigation. Full advancement of the catheter was identified by estimating the length of the catheter prior to insertion cranially. In addition, resistance to further advancement was felt when approaching the foramen magnum, and a clear effluent confirmed adequate advancement and irrigation. In contrast, full advancement in the caudal direction was confirmed by observing the tip of the catheter at the incision/laminectomy site of the subsequent incision inferiorly. Therefore, an intraoperative X-ray to confirm the catheter position was not required.

Midthoracic Incision

The catheter was then inserted epidurally through the midthoracic laminectomy (entry point) toward the cervical incision while maintaining irrigation. The EVD catheter was advanced superiorly until the tip of the catheter was observed through the cervical incision (exit point). Irrigation was continued until the effluent was clear. This ensured adequate evacuation of the abscess between the upper two incisions.

Lower Thoracic and Lumbar Incisions

**Figure 2:**
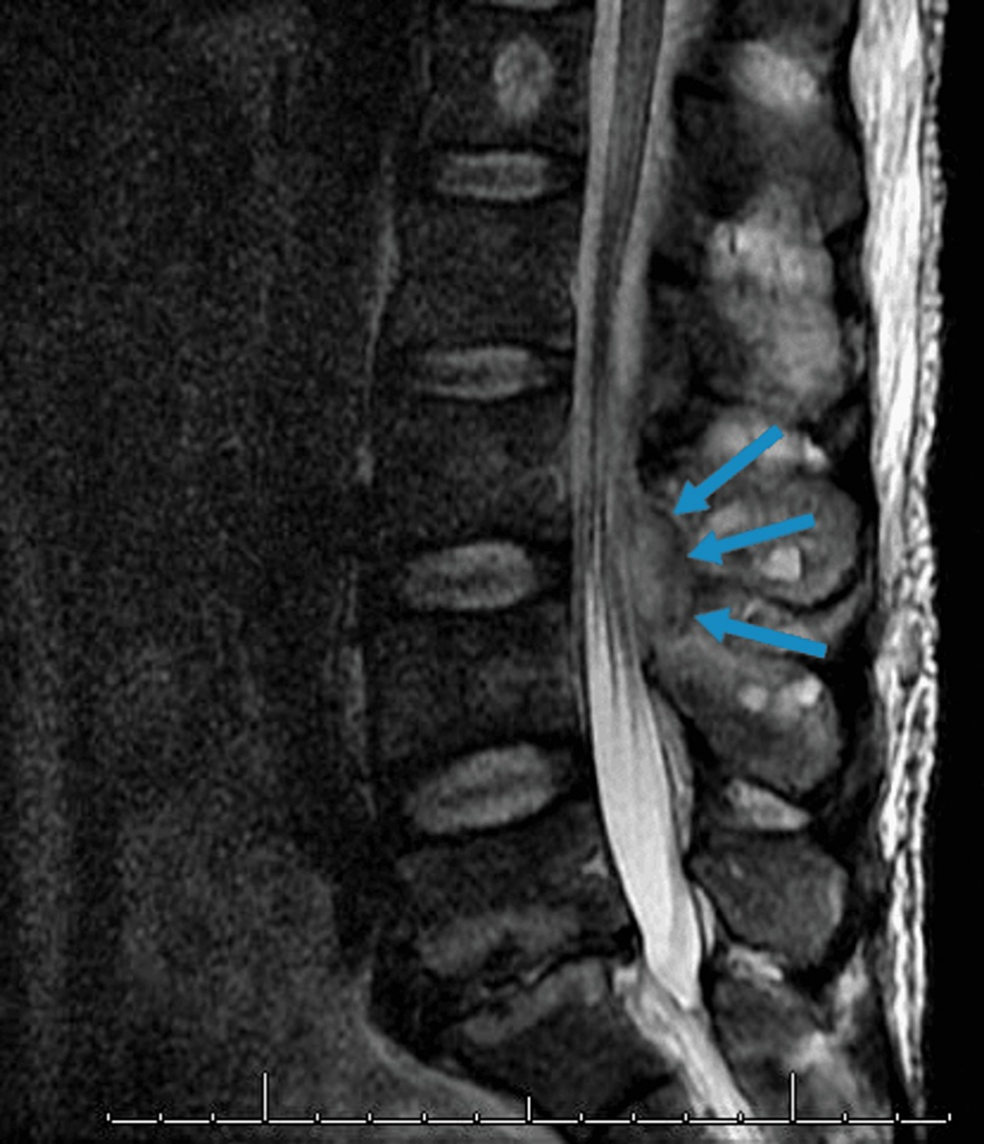
Adhesive collection at the lower spine T2-weighted sagittal MRI of the lumbar spine showing dorsal epidural collection adherent to the thecal sac at the lumbar spine (blue arrows) MRI: magnetic resonance imaging

The previous step was repeated during the lower thoracic and lumbar laminectomies. Care was taken to ensure adequate irrigation between the entry and exit points. Appropriate culture specimens were obtained. During the decompression of the lumbar spine, the left L3-L4 facet joints were found to be significantly deteriorated with purulent drainage from the joint (plus osteomyelitis and discitis at L3-L4 detected on preoperative imaging). Therefore, L3-L4 posterior instrumentation with pedicle screws and rods was performed (followed by anterior discectomy, interbody arthrodesis with allograft, and expandable cage placement in a second surgery one week later). In addition, a phlegmon adherent to the thecal sac (noticed preoperatively {Figure 2}) was carefully removed allowing for rapid decompression of the distal cord.

The debridement of the outer part of the phlegmon was first performed, allowing for easier access and resection of the inner part. This has also facilitated catheter irrigation and drainage of any liquified purulent material. Importantly, intraoperative neuromonitoring during cranial and caudal advancement of the catheter was used to avoid and monitor unnecessary injury to the already compressed spinal cord. Caudal irrigation of the sacral region was performed through the last incision (lumbar) toward the sacrum. All incisions were irrigated with normal saline until the effluent was clear. Hemovac drains were left in situ at each surgical wound before the closure of the fascia and skin. The patient was then transferred to the intensive care unit (ICU) for further monitoring and treatment.

Postoperative outcomes and follow-up

Pus and tissue cultures grew methicillin-sensitive *Staphylococcus aureus* that was resistant to clindamycin and erythromycin. Urine and blood cultures were also positive for *S. aureus*. The patient then received intravenous antibiotics for six weeks. On postoperative day 7, the patient regained sensory function in the lower extremities and some motor function in the upper extremities (two out of five). Two weeks after the surgery, he was able to move his upper extremities against gravity but with some difficulty (three out of five). Motor strength in his lower extremities was two out of five.

At the three-month outpatient follow-up, neurological examination showed improvement in motor strength (4/5 in upper extremities and 3+/5 in lower extremities). There were no postoperative complications, and the patient reported restoration of urinary continence. The sensory level was completely restored except for mild paresthesia in the feet. His clinical and functional improvement and the absence of complications indicated successful decompression and recovery. Follow-up MRI was therefore not necessary. During his hospital stay, the patient had an inpatient rehabilitation before discharging to outpatient and physical therapy facilities as recommended.

## Discussion

HEA is an exceedingly rare but severe infection of the spine, with only a small number of cases reported in the literature. It is typically seen in individuals with comorbid chronic conditions (such as diabetes mellitus, as seen in the current case), intravenous drug abuse, and immunocompromised states such as malignancy and chronic glucocorticoid use [1,8]. Other important risk factors include concurrent local and/or systemic infections and invasive procedures (e.g., epidural anesthesia and spinal procedures) [1]. Symptoms typically begin with localized pain over the spine of the affected area [8]. Due to the ability of this infection to spread along the spinal cord, it can rapidly progress to serious and permanent neurological deficits (e.g., radiculopathy, compromised bowel and bladder function, and progressive weakness) and even death. Therefore, timely diagnosis and management are crucial to prevent vascular compression (from the abscess and associated inflammatory edema) and to allow for better surgical outcomes [9].

The evidence of clinical and radiographic spinal cord compression and/or ischemia (e.g., worsening neurological symptoms) warrants emergent surgical evacuation along with empiric broad-spectrum antibiotics [10]. Both blood and intraoperative tissue cultures are useful for identifying the organism, with *S. aureus* being the most common causative pathogen [1,8]. For cases in which the organism cannot be identified, empiric antibiotic treatment is recommended. In the present case, the patient developed deteriorating extremity weakness with notable spinal cord compression on spine MRI, requiring emergent surgical decompression.

The goal of HEA surgical treatment is to decompress the spinal cord and adequately eliminate the source of infection. Due to the extension of the abscess along the entire spinal cord, skip laminectomies at multiple inconsequent levels are usually preferred by most authors [1-7] to both provide sufficient drainage and preserve the tension provided by the posterior ligamentous complex, which normally resists kyphotic forces. The consensus on the appropriate technique for adequate drainage of the abscess and catheter irrigation remains undecided. In the current case, a single laminectomy through each incision was performed (except for the L2-L4 incision) to maintain mechanical stability. Catheter irrigation was then carried out caudally and cranially through these laminectomies, minimizing the need for contiguous laminectomies and later excessive instrumentation.

Following the removal of the laminae, intraoperative drainage of the abscess and epidural catheter irrigation of the liquified pus must be performed. This particular step is essential because it allows for successful decompression and complete elimination of infection along with antibiotics. At this point, however, the surgeon performs blind evacuation and relies heavily on catheter irrigation to ensure surgical decompression and clearance of the purulent material. Denis et al. [11] suggested the use of intraoperative fluoroscopy for the proper placement of radiopaque epidural catheters to enhance the safety and efficacy of the irrigation process. Alternatively, Khattar et al. [2] described the use of omnipaque intraoperative dye to ensure the complete evacuation of the entire abscess and to visualize any septations that may prevent further drainage. Intraoperative X-ray images were also used to locate the extent of catheter advancement. These techniques have their own risks and limitations. Besides the risks of intraoperative radiation to staff and patients, these techniques do not allow for direct visualization of the abscess; however, they instead visualize the radiopaque catheter during advancement [2,11]. Although the use of omnipaque intraoperative dye allows for the verification of the removal of the infection foci, the presence of septations or complex abscesses poses limitations due to incomplete dye intake [2]. In our case, intraoperative imaging was not required because the tip of the catheter was further advanced until observed from the adjacent incision. This facilitated access to distant perispinal spaces and optimized decompression of the spinal cord, conferring little to no additional risk.

An open surgical approach improves direct visualization of the abscess but increases tissue disruption and may precipitate postoperative pain. Minimally invasive techniques offer superior safety and less tissue damage [12,13]. Although they are associated with limited visualization of the abscess compared to open laminectomy, they have comparable efficacy. In our patient, we utilized the mini-open approach, allowing for optimal decompression of the spinal cord, less iatrogenic instability and tissue disruption, decreased blood loss, and adequate irrigation of abscess.

In most reported studies in the literature, the catheter was advanced caudally and cranially from one incision without a predetermined destination or observing the extent of catheter advancement in the epidural space [4,5,11,14-17]. As a result, there is a risk of incomplete drainage of the abscess or inadequate irrigation of distant and hidden sites around the spinal cord. Moreover, this may place the already compromised spinal cord at risk for additional damage, especially when using non-flexible catheters such as the Fogarty embolectomy catheter [18]. In our case, the observation of the terminal point of catheter advancement optimized debridement without the need for intraoperative guidance. As the surgeon approaches the adjacent incision during irrigation, the displacement of purulence can be observed at the exit point of the catheter. The amount and flow of purulence at the exit point indicate the proximity of the catheter tip to the incision, providing a tool for tracking the catheter and estimating its location along the length of the spinal cord.

Finally, pus typically contains living bacteria that may further replicate if not properly washed out. We believe that any remaining liquified or conglomerated pus in the affected area may play a role in sustaining the survival of the offending organism and the regeneration of the abscess, especially in the setting of highly resistant microorganisms and immunocompromised patients. This may present as a worsening of neurological function or failure to improve following the surgery, as seen in some cases in the literature [14,15,19]. Recurrence is usually demonstrated on imaging (e.g., MRI) and appears as focal spinal compression in the area with the highest infectious burden (probably obscured and distant sites from the tip of the catheter).

## Conclusions

HEA patients who present with deteriorating neurological deficits require surgical intervention to prevent spinal cord ischemia and subsequent permanent damage. Skip laminectomies are usually performed in conjunction with irrigation and aspiration of purulent material. Epidural catheter irrigation is an essential step in the emergent surgical evacuation of HEA. Tunneling and connecting the catheter tip epidurally between the incisions while performing irrigation ensure complete debridement of the abscess in distant and hidden sites around the spinal cord. In addition, it minimizes the number of contiguous laminectomies and associated instrumentation.
